# Evaluation and comparison of FTA card and CTAB DNA extraction methods for non-agricultural taxa^1^

**DOI:** 10.3732/apps.1600109

**Published:** 2017-02-15

**Authors:** Chloe S. Siegel, Florence O. Stevenson, Elizabeth A. Zimmer

**Affiliations:** 2 Department of Crop Sciences: Plant Biotechnology and Molecular Biology, University of Illinois at Urbana-Champaign, 1102 S. Goodwin Avenue, Urbana, Illinois 61801 USA; 3 Department of Mechanical Engineering, Department of Mathematics, University of Maryland, 2181 Glenn L. Martin Hall, Building 088, College Park, Maryland 20742 USA; 4 Department of Botany, Smithsonian Institution National Museum of Natural History, NMNH-MRC, 166 Smithsonian Institution, P.O. Box 37012, Washington, D.C. 20013-7012 USA

**Keywords:** CTAB-based technique, DNA extraction, amplification, and sequencing, fluorometry, FTA cards, gel electrophoresis, spectrophotometry

## Abstract

**Premise of the study:**

An efficient, effective DNA extraction method is necessary for comprehensive analysis of plant genomes. This study analyzed the quality of DNA obtained using paper FTA cards prepared directly in the field when compared to the more traditional cetyltrimethylammonium bromide (CTAB)–based extraction methods from silica-dried samples.

**Methods:**

DNA was extracted using FTA cards according to the manufacturer’s protocol. In parallel, CTAB-based extractions were done using the automated AutoGen DNA isolation system. DNA quality for both methods was determined for 15 non-agricultural species collected in situ, by gel separation, spectrophotometry, fluorometry, and successful amplification and sequencing of nuclear and chloroplast gene markers.

**Results:**

The FTA card extraction method yielded less concentrated, but also less fragmented samples than the CTAB-based technique. The card-extracted samples provided DNA that could be successfully amplified and sequenced. The FTA cards are also useful because the collected samples do not require refrigeration, extensive laboratory expertise, or as many hazardous chemicals as extractions using the CTAB-based technique.

**Discussion:**

The relative success of the FTA card method in our study suggested that this method could be a valuable tool for studies in plant population genetics and conservation biology that may involve screening of hundreds of individual plants. The FTA cards, like the silica gel samples, do not contain plant material capable of propagation, and therefore do not require permits from the U.S. Department of Agriculture (USDA) Animal and Plant Health Inspection Service (APHIS) for transportation.

Successful extraction of DNA from plant tissues generally has been most successful from freshly field-collected tissue placed into either liquid nitrogen or, more commonly, into silica gel desiccant. When possible, the samples are then stored frozen in ultracold liquid nitrogen tanks or in boxes containing additional silica gel at −20°C to −80°C. Subsequent nucleic acid extractions most commonly have involved either some form of a cetyltrimethylammonium bromide (CTAB)–based technique (Doyle and Doyle, 1987) or use of a QIAGEN DNeasy kit (QIAGEN, Venlo, The Netherlands). However, previous research has also shown success with an alternative genomic DNA extraction method using Whatman FTA PlantSaver Cards (Whatman, Maidstone, United Kingdom), with effectiveness for agricultural plant species, entomology, mycology, and other food sciences (Suzuki et al., 2006; Marques et al., 2010; Adugna et al., 2011; Bujang et al., 2011; Chandrashekara et al., 2012). Nevertheless, remarkably little research has been done to test the effectiveness of this extraction method on non-agricultural plant species. Additionally, it has been shown that methods employing DNeasy kits and/or CTAB-based methods may not be optimal for all plant genera and families because inhibitors can be coprecipitated with the DNA (Bustin and Nolan, 2004; Adugna et al., 2011). Therefore, it is proposed that alternative extraction methods, such as use of the FTA cards, be tested as to whether they are more effective for some plant genera.

To help gain a better understanding of the potential uses and limitations of FTA card extraction, this study assessed FTA card–extracted DNA quality in terms of concentration, spectral absorption, degree of fragmentation, and amplification and sequencing ability on a wide phylogenetic range of non-agricultural species. The data collected using these methods helped to indicate which plant species may be most compatible with the FTA card extraction method. Both successful and failed extractions provided valuable insights into the potential advantages and limitations of this alternative extraction method.

## MATERIALS AND METHODS

### Collection

Samples from 15 phylogenetically diverse taxa possessing varying leaf characteristics were collected from the following vascular plant families: Apocynaceae, Aquifoliaceae, Asteraceae, Cactaceae, Cyperaceae, Fabaceae, Lamiaceae, Magnoliaceae, Oleaceae, Oxalidaceae, Poaceae, Typhaceae, Vitaceae, Pinaceae, and Aspleniaceae (Table 1). Collection of samples was completed on the morning of 23 June 2015, alongside a gravel utility road, sloping hillsides, and free-standing trees in Suitland, Maryland, USA. Voucher specimens for each species were deposited in the National Museum of Natural History’s Herbarium (US), the University of Illinois at Urbana-Champaign Plant Biology Herbarium (ILL), and the Chicago Botanic Garden (CHIC).

**Table 1.** Species sampled in this study. All specimens were collected in Camp Springs, Maryland, USA (GPS coordinates 38°50′40.4″N, 76°56′17.4″W). Triplicate vouchers were made for deposit at the National Museum of Natural History’s Herbarium (US), the University of Illinois at Urbana-Champaign Plant Biology Herbarium (ILL), and the Chicago Botanic Garden (CHIC). Samples are organized alphabetically by family name.

Family	Genus/Species (Common name)	Voucher no.
Aquifoliaceae	*Ilex glabra* (L.) A. Gray (inkberry)	*C. Siegel 11*
Asclepiadaceae	*Asclepias syriaca* L. (common milkweed)	*C. Siegel 10*
Aspleniaceae	*Asplenium platyneuron* (L.) Britton, Sterns & Poggenb. (ebony spleenwort)	*C. Siegel 6*
Asteraceae	*Ratibida pinnata* (Vent.) Barnhart (pinnate prairie coneflower)	*C. Siegel 8*
Cactaceae	*Opuntia* cf. *laevis* J. M. Coult. (prickly pear)	*C. Siegel 15*
Cyperaceae	*Carex lurida* Wahlenb. (shallow sedge)	*C. Siegel 3*
Fabaceae	*Albizia julibrissin* Durazz. (silktree)	*C. Siegel 7*
Lamiaceae	*Monarda fistulosa* L. (wild bergamot)	*C. Siegel 1*
Magnoliaceae	*Magnolia virginiana* L. (sweetbay magnolia)	*C. Siegel 12*
Oxalidaceae	*Oxalis dillenii* Jacq. (wood sorrel)	*C. Siegel 13*
Pinaceae	*Pinus virginiana* Mill. (Virginia pine)	*C. Siegel 5*
Poaceae	*Dichanthelium commutatum* (Schult.) Gould (panicgrass)	*C. Siegel 14*
Simaroubaceae	*Ailanthus altissima* (Mill.) Swingle	*C. Siegel 9*
Typhaceae	*Typha angustifolia* L. (narrowleaf cattail)	*C. Siegel 2*
Vitaceae	*Ampelopsis glandulosa* (Wall.) Momiy. (Amur peppervine)	*C. Siegel 4*

**Fig. 1.**
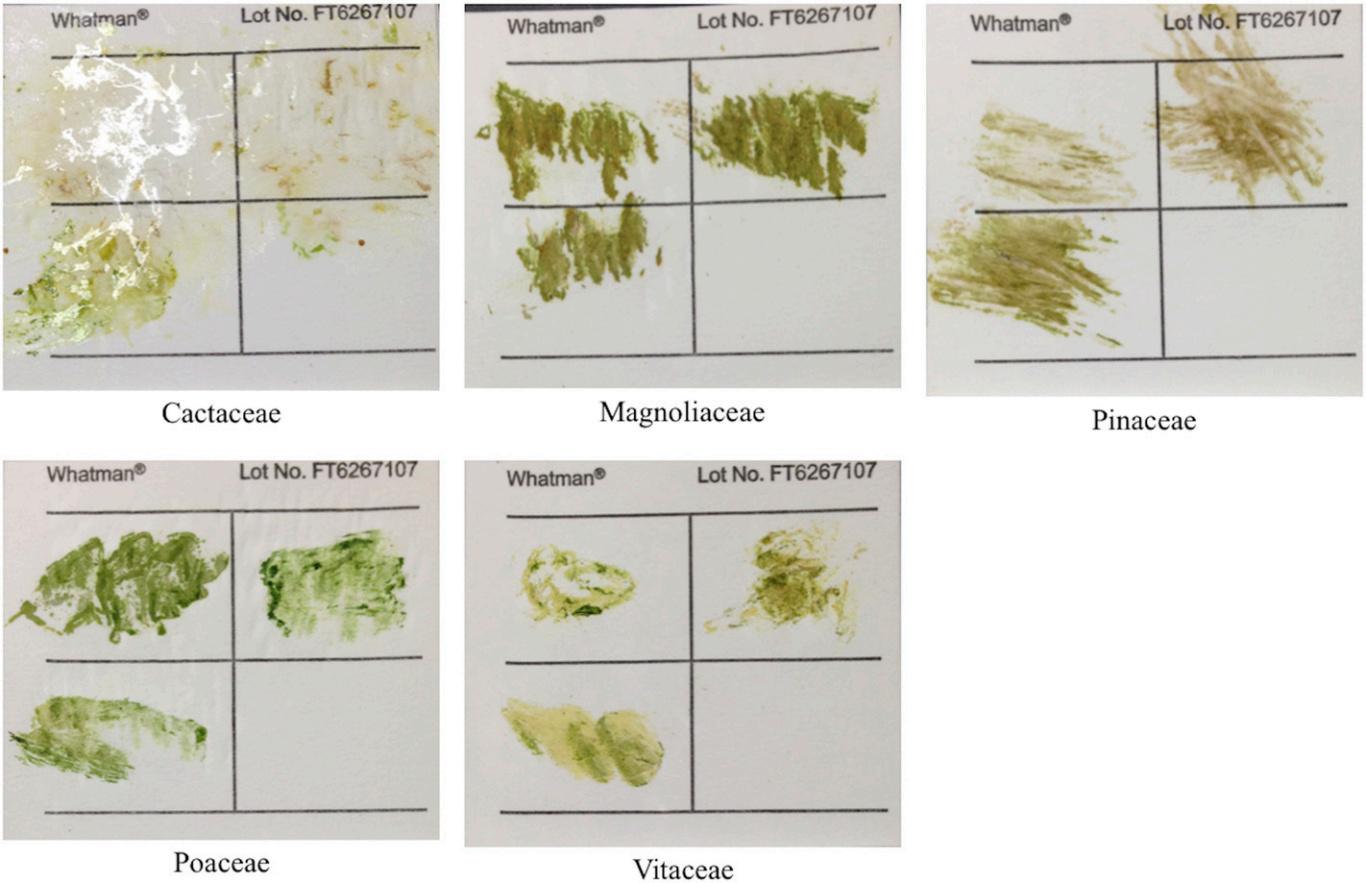
Whatman FTA PlantSaver card samples, pre-extraction. The four quadrants of several FTA cards covering the phylogenetic range of the sampled species are shown. Three quadrants of each card were used to press leaf tissue. Eight hole-punches were obtained for each well of a 96-well plate.

Samples were preserved using Whatman FTA PlantSaver Cards and in silica gel (Flower Drying Type A silica with indicator; AGM Container Controls, Tucson, Arizona, USA). Samples were applied to the FTA cards directly in the field, then stored at room temperature. To make the plant print, a ceramic pestle was used like a hammer to smash the leaf tissue onto the card paper (Fig. 1). Enough plant prints were made for seven replicate extractions to be performed for each species.

Additionally, 2–3 in^2^ of leaf tissue were collected into individual silica gel–containing bags to be extracted later, using the CTAB-based technique on the AutoGen DNA isolation system (AutoGen, Holliston, Massachusetts, USA). Bags were stored in a plastic box containing additional silica gel at room temperature prior to extraction and then moved to a −20°C freezer for long-term storage.

### Extraction

#### FTA card extraction

After collecting the samples on the FTA cards, small punched disks were removed from the sample cards followed by a series of washes on the disks. The protocol used was closely modeled after the method outlined in Adugna et al. (2011), with a modified disk size and centrifugation added after the incubation period to reduce bubbles. Eight disks, 2.0 mm in diameter, were used in this study so as to have a comparable total disk surface area to that found in Adugna et al. (2011). Additionally, the protocol of Adugna et al. called for centrifugation only after the addition of TE in the last step of the protocol. In our study, plates were centrifuged at 6200 rpm for 2 min at 4°C in between each purification wash.

Using the 2.0-mm-diameter Harris Micro Punch and Mat (Whatman), 56 disks were removed from each specimen’s plant-pressed FTA card. A 96-well, Square V-Bottom 2-mL Assay Block (Costar, Corning Inc., Corning, New York, USA) was used with eight punched disks placed in each well. The disks were only added to the innermost wells (rows 3–10 out of 12) of the plates for more effective and cleaner transfer from one plate to another. Disk punches were taken from the leaves’ darker print areas on the cards, which were presumed to contain the most concentrated residue.

Once disks had been collected from each plant, a series of washes were employed according to the manufacturer’s protocol. First, 400 μL of FTA purification reagent was added to each well. The plate of samples was then covered with Microseal ‘F’ foil seals (Bio-Rad, Hercules, California, USA) to reduce contamination, vortexed, and incubated at room temperature for 4 min before centrifugation at 6200 rpm for 2 min at 4°C. The supernatant was removed and the plates were then centrifuged a second time before the foil was removed. The FTA purification reagent wash was repeated once, followed by two low TE buffer washes. After completing the four washes, the remaining supernatant was removed. The samples were left to dry at room temperature for 20 min. The disks were then transferred to a new plate and centrifuged to separate them from as much remaining supernatant as possible. Any punches not in the new plate were manually transferred using cleaned forceps. Finally, 80 μL of TE was added to the plate containing the washed disks. The plate was centrifuged at 6200 rpm for 1 min and then incubated for 5 min at 95°C. The paper disks were left in the sample wells with the TE and eluted DNA.

#### CTAB-based extraction

For each replicate, a 1.0-cm^2^-sized piece of dried and shredded leaf tissue was added to a 2.0-mL tube containing 2.3-mm-diameter zirconia-silica beads and 1.0-mm-diameter glass beads (BioSpec, Bartlesville, Oklahoma, USA). The tissue was then macerated using the TissueLyser (QIAGEN) at 30 Hz for 30 s before 400 μL of CTAB, warmed to 65°C, was added. The Cactaceae samples produced a viscous gel, so 75 μL of 20 mg/mL Proteinase K and 500 μL of CTAB were added to further clean the sample. All samples were incubated overnight in a rotary incubator at 65°C and 150 rpm.

The resulting lysate solutions were centrifuged at 13,000 rpm for 5 min and 300 μL of supernatant was transferred to each well of a 96-well AutoGen plate. DNA extraction was completed using the automated DNA isolation system AutoGenprep 965 (AutoGen) as outlined by the manufacturer’s protocol, and the final pellets were resuspended in 80 μL of TE buffer.

### Quantification and quality assessment

**Fig. 2.**
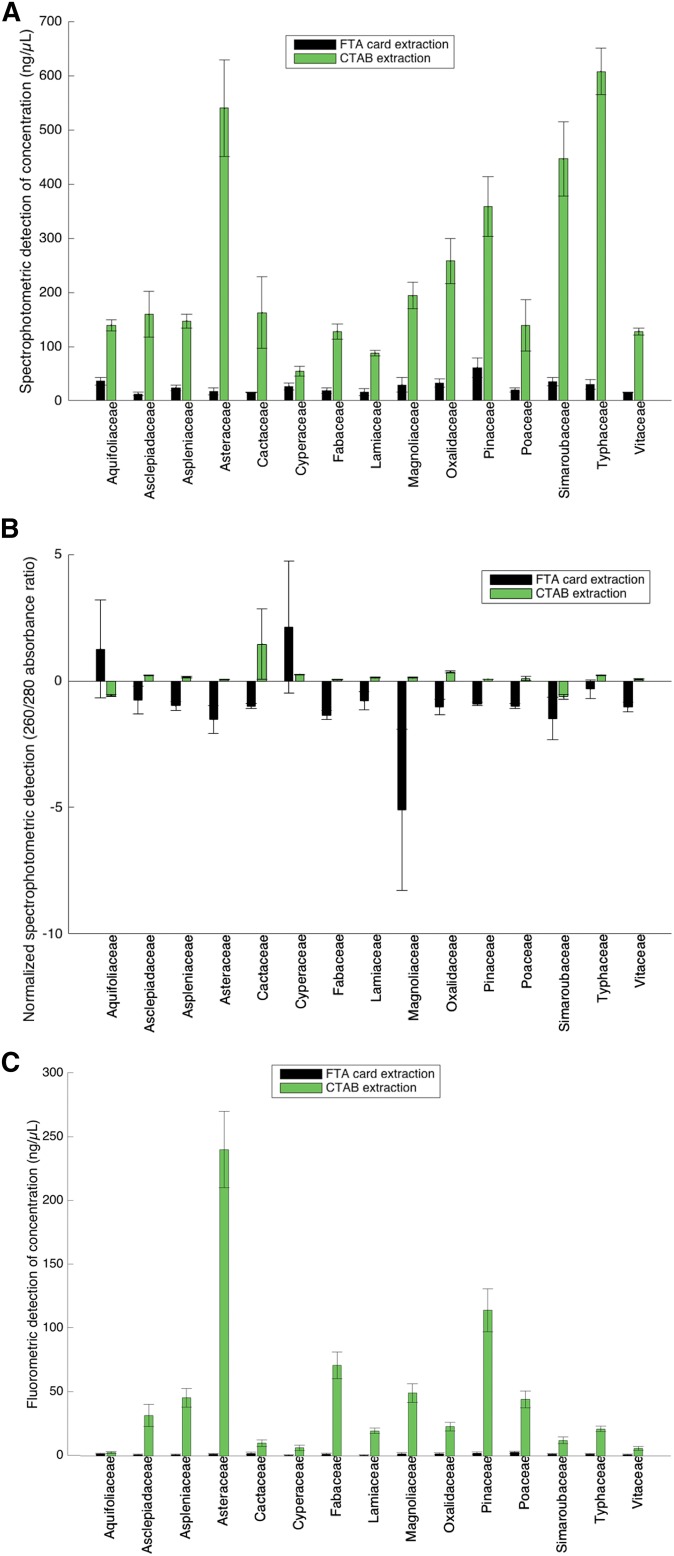
DNA concentration and quality in plant extractions. (A) Concentration (ng/μL) **±** 1 SE as detected by the Epoch Spectrophotometer System determined for seven replicates. Nucleic acid concentration was indicated by absorbance measured at 260 nm. Measurements did not discriminate between RNA and DNA concentrations. (B) 260/280 absorbance ratio as detected by the Epoch Spectrophotometer System. Absorbances measured at 260 nm and 280 nm with suboptimal 1.8 absorbance ratios indicated the presence of contaminants. Detected values have been normalized to 0 by subtracting 1.8 from each measured ratio value. (C) Concentration (ng/μL) as detected by the Quant-iT Fluorometer. Fluorescence-based dyes were bound to the DNA in each sample. This technique does discriminate between RNA and DNA.

**Fig. 3.**
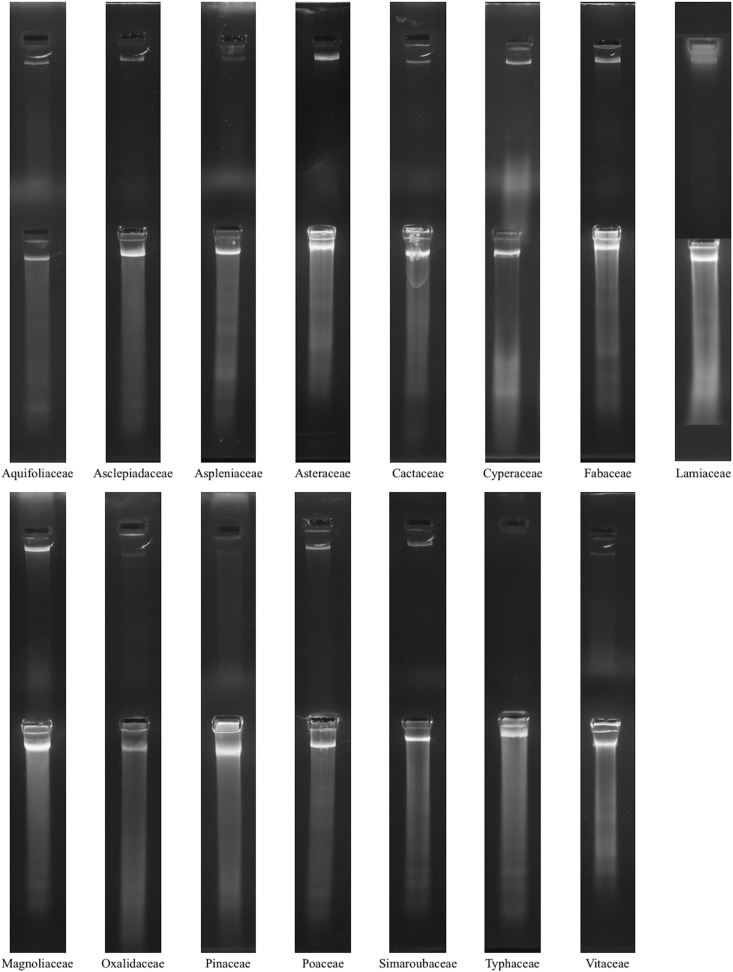
Total genomic DNA extract gel electrophoresis results. The FTA card–extracted DNA is seen in the top row while the CTAB-extracted DNA is seen in the bottom row. All seven replicates of each plant extraction method were run on the gel, but only one replicate per species is shown.

To assess the quality of the extractions generated with the two techniques, 260/280 nm absorbance ratios and fluorometric determinations of DNA concentration were performed using a Synergy HT Microplate Reader (BioTek, Winooski, Vermont, USA). The Quant-iT Broad-Range dsDNA Assay Kit (Invitrogen, Waltham, Massachusetts, USA) was used with the Synergy Microplate Reader according to the manufacturer’s protocol (Fig. 2). The DNA size range and degree of fragmentation were determined using gel electrophoresis (Fig. 3). The samples (10 μL) were run alongside a HiLo DNA size standard (Minnesota Molecular, Minneapolis, Minnesota, USA) on a 1.5% SeaKem Agarose LE gel in 1× SB (Brody and Kern, 2004). The loading dye used to prepare these electrophoretic separations contained a 1:1000 dilution of Gel Red fluorescent nucleic acid stain (Biotium, Fremont, California, USA).

### PCR amplification and DNA sequencing

To compare the amplification success of DNA extracted with the two methods, a portion of the low-copy nuclear gene *At103*, the nuclear ribosomal intergenic spacer (ITS), and the plastid ribulose-bisphosphate-dismutase large subunit (*rbcL*) were amplified. Each amplification reaction contained 2.5 μL of FTA card–extracted sample or 2.5 μL of 1:50 diluted CTAB-extracted sample. The *At103* region was amplified with forward primer CTTCAAGCCMAAGTTCATCTTCTA and reverse primer TTGGCAATCCATTGAGGTACATNGTM (Li et al., 2008), and the ITS region was amplified with primers ITS5a (CCTTATCATTTAGAGGAAGGAG) and ITS4 (CAGGAGACTTGTACACGGTCCAG; Kress et al., 2005). For these primer pairs, the PCR program used was: 95°C for 4 min; four cycles at 95°C for 45 s, 57°C for 30 s, 72°C for 1.5 min; four cycles at 95°C for 45 s, 54°C for 30 s, 72°C for 1.5 min; 35 cycles at 95°C for 45 s, 52°C for 30 s; 72°C for 1.5 min; and a final extension at 72°C for 10 min. For *rbcL*, PCR was conducted with forward primer ATGTCACCACAAACAGAGACTAAAGC and reverse primer GTAAAATCAAGTCCACCRCG (Kress et al., 2005) using the following thermal cycler protocol: 95°C for 3 min; 34 cycles at 94°C for 30 s, 55°C for 45 s, 72°C for 2 min; and a final extension at 72°C for 5 min. Success of the PCR was assessed by gel electrophoresis.

Two successful replicates for each marker and species were chosen for sequencing. These products were enzyme-treated with ExoSAP-IT (Affymetrix, Santa Clara, California, USA) to remove unincorporated primer and deoxynucleotide triphosphates (dNTP) prior to being used as template for cycle sequencing with BigDye Terminator version 3.1 Ready Reaction Mix (Thermo Fisher Scientific, Grand Island, New York, USA). The resulting Sanger sequencing fragments were purified through Sephadex G-50 and analyzed on an ABI 3730xl automated capillary sequencer (Life Technologies, Carlsbad, California, USA). Chromatograms were analyzed using Geneious 9.0.5 (Biomatters, Auckland, New Zealand), and successful sequences were submitted to GenBank (Table 2).

**Table 2.** Results of DNA amplifications and sequencing.^a^

Family	Marker	Extracted using CTAB method				Extracted using FTA method			
		No band	>1 band	1 band	GenBank accession no.^b^	No band	>1 band	1 band	GenBank accession no.^b^
Aquifoliaceae	*At103*	0	7	0	No	0	7	0	KX394243
	ITS	0	0	**7**	KX352749	0	0	**7**	KX352750
	*rbcL*	4	0	**3**	—	0	7	0	KX702293
Asclepiadaceae	*At103*	4	2	**1**	No	7	0	0	No
	ITS	0	7	0	KX352744	0	7	0	KX352745
	*rbcL*	0	0	**7**	KX702286	0	0	**7**	KX702287
Aspleniaceae	*At103*	6	0	**1**	KX394240	7	0	0	—
	ITS	0	5	**2**	No	1	1	**5**	No
	*rbcL*	0	0	**7**	KX702288	2	0	**5**	KX702289
Asteraceae	*At103*	2	5	0	No	3	1	**3**	No
	ITS	0	0	**7**	KX352753	0	0	**7**	KX352754
	*rbcL*	0	0	**7**	KX702303	0	5	**2**	KX702304
Cactaceae	*At103*	0	7	0	KX394245	0	0	**7**	KX394246
	ITS	0	7	0	KX352751	1	6	0	KX352752
	*rbcL*	1	0	**6**	KX702298	0	7	0	KX702299
Cyperaceae	*At103*	7	0	0	—	6	0	**1**	No
	ITS	1	1	**5**	No	0	0	**7**	KX352746
	*rbcL*	0	6	**1**	KX702290	0	2	**5**	KX702291
Fabaceae	*At103*	0	7	0	KX394236	0	7	0	KX394237
	ITS	0	6	**1**	No	0	7	0	No
	*rbcL*	0	2	**5**	KX702282	0	1	**6**	KX702283
Lamiaceae	*At103*	0	0	**7**	KX394244	6	1	0	No
	ITS	1	4	**2**	No	7	0	0	—
	*rbcL*	0	7	0	KX702296	4	0	**3**	KX702297
Magnoliaceae	*At103*	7	0	0	—	5	2	0	No
	ITS	0	7	0	—	0	7	0	—
	*rbcL*	0	5	**2**	KX702295	0	7	0	KX702294
Oxalidaceae	*At103*	0	7	0	KX394247	1	5	**1**	KX394248
	ITS	0	0	**7**	No	0	1	**6**	No
	*rbcL*	0	4	**3**	KX702300	1	6	0	KX702301
Pinaceae	*At103*	0	7	0	No	7	0	0	—
	ITS	0	5	**2**	No	7	0	0	—
	*rbcL*	0	0	**7**	KX702302	7	0	0	—
Poaceae	*At103*	0	7	0	KX394241	3	3	**1**	KX394242
	ITS	0	7	0	KX352747	0	7	0	KX352748
	*rbcL*	0	4	**3**	No	2	5	0	KX702292
Simaroubaceae	*At103*	3	4	0	KX394234	0	0	**7**	KX394235
	ITS	2	3	**2**	No	0	7	0	KX352741
	*rbcL*	3	0	**4**	KX702280	0	7	0	KX702281
Typhaceae	*At103*	0	0	**7**	KX394249	3	0	**4**	KX394250
	ITS	0	6	**1**	No	0	7	0	No
	*rbcL*	0	4	**3**	KX702305	2	1	**4**	No
Vitaceae	*At103*	0	0	**7**	KX394238	4	0	**3**	KX394239
	ITS	1	1	**5**	KX352742	0	5	**2**	KX352743
	*rbcL*	0	0	**7**	KX702284	4	3	0	KX702285

a Families are listed alphabetically with the number of replicates (out of a total of seven) that showed no bands, multiple bands, or one band. The number of replicates that were successfully amplified with a single band are provided in boldface. Plant samples were amplified with three different primer pairs: ITS, *rbcL*, and *At103*.

b — = PCR was not successful and, as a result, sequencing was not performed; No = sequencing was performed on an amplicon, but was not successful. GenBank accession numbers are provided for amplicons for which PCR and sequencing were successful.

## RESULTS

### Quantification and quality assessment

Spectrophotometric estimations of nucleic acid concentrations of CTAB-extracted samples were considerably higher than those of FTA card–extracted samples (Fig. 2A). However, there was not a substantial difference in average detected concentration among the 15 sampled species. Although the CTAB-extracted samples were much more concentrated, the CTAB sample concentration values were more variable than those of the FTA card–extracted samples. FTA card samples had an average standard error for concentration of 7.81 ng/μL while that of the CTAB-extracted samples was 37.96 ng/μL. The average 260/280 absorbance ratio was closer to 1.8 for CTAB-extracted samples compared to those for FTA card–extracted samples (Fig. 2B). The FTA card–extracted, amplified samples also had 260/280 ratios that were indicative of lower quality.

Quant-iT fluorometry showed that the CTAB-extracted samples contained large, but highly variable concentrations of DNA, while the DNA extracted with the FTA cards was relatively undetectable with this method (Fig. 2C). Like the spectrophotometric measurements, there were no substantial differences in average concentration among the 15 sampled species. Because spectrophotometry does not discriminate between RNA and DNA as effectively as the fluorometric method, the greater quantities of DNA estimated by the spectral absorbance values (Fig. 2A vs. Fig. 2C) might be an artifact of the copurified RNA with the DNA in the samples.

Gel separations of the samples indicate that all of these total genomic DNA extracts contained unfragmented, high-molecular-weight DNA, except for the Typhaceae samples extracted with the FTA card method (Fig. 3).

### PCR amplification and DNA sequencing

CTAB and FTA card samples for all families were generally successful in amplification of *At103*, ITS, and *rbcL* (Table 2). Despite the differences seen in the appearance of each plant print on the FTA cards (Fig. 1), all but the conifer *Pinus virginiana* could be successfully amplified with at least one primer pair (Table 2). The *rbcL* amplicons were the most amenable to cycle sequencing, resulting in high-quality chromatograms for at least one CTAB and/or FTA card sample of each species (Table 2). The *At103* marker most readily amplified and sequenced for families Aquifoliaceae, Aspleniaceae, Cactaceae, Fabaceae, Lamiaceae, Oxalidaceae, Poaceae, Simaroubaceae, Typhaceae, and Vitaceae. The most successful families for amplification and sequencing with the ITS5a/ITS4 primers were Aquifoliaceae, Asclepiadaceae, Asteraceae, Cactaceae, Cyperaceae, Poaceae, Simaroubaceae, and Vitaceae. These results indicated that, even if the leaf did not create a dark chlorophyll print, amplifiable DNA was captured by the FTA card.

## DISCUSSION

The FTA card method could have great utility in the study of non-agricultural plant phylogenetics and population genetics, as it addresses some shortcomings of the CTAB-based technique, including facility of collection and transportation. During field collection, kilogram quantities of silica would not need to be transported to preserve specimens for CTAB-based extraction methods, allowing more specimens to be collected. While collection permits are usually required for sampling plants, a U.S. Department of Agriculture Animal and Plant Health Inspection Service (USDA-APHIS) permit is not necessary for acquiring the actual plant tissue embedded on an FTA card, because the material cannot be propagated, as would whole-rooted plants, rhizomes, and seeds (V. Funk, personal communication). Additionally, because the FTA card embedding procedure may destroy RNA from viruses (Kraus et al., 2011), biosafety issues may not arise when transporting plant tissues between states and between countries. The FTA cards are more compactly stored and do not require refrigeration. Finally, the FTA card–extraction method requires less laboratory expertise and fewer hazardous chemicals such as CTAB, chloroform, and phenol (Suzuki et al., 2006; Marques et al., 2010; Chandrashekara et al., 2012).

The FTA card and CTAB extraction methods both exhibited varying levels of success. CTAB-extracted samples contained higher concentrations of DNA as estimated by their A260/A280 absorbance ratios. Between replicates of the same species there was greater variation in concentration among those extracted with the CTAB-based vs. the FTA card methods. This inconsistent quantity of DNA recovered from the CTAB procedure may have been an artifact of the AutoGen instrument, as has been previously reported (Mulcahy et al., 2016).

The increased concentrations of DNA detected in the CTAB-extracted samples is a major consideration in determining the overall utility of the two methods. The differences in DNA concentration for CTAB- and FTA card–extracted samples could have been due to the differences in amount of leaf tissue originally used for extraction. Approximately 1 cm^2^ of leaf tissue was used for each CTAB-extracted sample, while for FTA card–extracted samples the amount of DNA produced depended on the concentration and amount of fluid transferred from the leaves to the card.

The total genomic DNA gels (Fig. 3) indicated that the FTA card–extracted samples contained a less-fragmented DNA when compared to the CTAB-extracted samples. In contrast to the FTA card–extracted DNA, it was observed that RNA and/or degraded DNA could easily be detected in gel separations of the CTAB-extracted DNA.The amount of PCR product generated was approximately correlated with the concentration of the DNA extract used. Although many of the amplification success rates were similar between the two extraction methods, the CTAB-extracted samples on average amplified more consistently (Table 2). Only the Cactaceae, Cyperaceae, and Simaroubaceae species were amplified more successfully with the FTA card–extracted samples. In addition to the conifer and fern species, the families of the species that successfully amplified and sequenced were distributed evenly throughout the Angiosperm Phylogeny Group (APG) III phylogeny for flowering plants (Angiosperm Phylogeny Group, 2009; Tables 1, 2).

In summary, this study provided a more comprehensive understanding of the strengths and weaknesses of using Whatman FTA cards for non-agricultural species. Although the FTA card–extracted samples had a substantially lower concentration of DNA, the method is a good alternative for field collection if well-optimized primer pairs are being used that can amplify low concentrations of template DNA.

The previous success of FTA cards with large sample sizes in agricultural studies indicates that it could be a suitable method for studies in plant population genetics and conservation biology that require the analysis of hundreds to thousands of samples (Adugna et al., 2011; Chandrashekara et al., 2012). The FTA card method may work better for some families than for others. For example, we believe the FTA card method may work well for those families and genera phylogenetically related to and/or physiologically similar to the Cactaceae and Pinaceae.

Finally, while FTA cards have been used previously for extracting insect nucleic acids for RNA-Seq next-generation sequencing studies (Grubaugh et al., 2015), future work should focus on whether plant DNA and/or RNA extracted with the FTA cards could be used for some next-generation sequencing techniques (i.e., RNA-Seq, and possibly RAD-Seq, Genotyping by Sequencing [GBS], and targeted enrichment; Zimmer and Wen, 2015). By increasing the number and size of punches produced from the cards, FTA card–extracted samples might provide enough material suitable for the latter DNA-based next-generation sequencing methods.
